# Dynamic Contrast-Enhanced Magnetic Resonance Imaging (DCE-MRI) in Preclinical Studies of Antivascular Treatments

**DOI:** 10.3390/pharmaceutics4040563

**Published:** 2012-11-07

**Authors:** Thomas Nielsen, Thomas Wittenborn, Michael R. Horsman

**Affiliations:** Department of Experimental Clinical Oncology, Aarhus University Hospital, DK-8000 Aarhus C, Denmark; Email: wittenborn@oncology.dk (T.W.); mike@oncology.dk (M.R.H.)

**Keywords:** DCE-MRI, angiogenesis inhibitors, vascular disrupting agents, preclinical studies

## Abstract

Antivascular treatments can either be antiangiogenic or targeting established tumour vasculature. These treatments affect the tumour microvasculature and microenvironment but may not change clinical measures like tumour volume and growth. In research on antivascular treatments, information on the tumour vasculature is therefore essential. Preclinical research is often used for optimization of antivascular drugs alone or in combined treatments. Dynamic contrast-enhanced magnetic resonance imaging (DCE-MRI) is an *in vivo* imaging method providing vascular information, which has become an important tool in both preclinical and clinical research. This review discusses common DCE-MRI imaging protocols and analysis methods and provides an overview of preclinical research on antivascular treatments utilizing DCE-MRI.

## 1. Introduction

### 1.1. Tumour Microvasculature and Antivascular Treatments

It is now well established that for virtually all solid tumours to grow they must develop their own functional blood supply [1,2]. They do this from the normal host vessels by the process of angiogenesis [3,4]. Once established, the neo-vasculature not only supplies tumour cells with essential oxygen and nutrients [5], it is also the principal vehicle for metastatic spread [6]. However, the neo-vasculature formed is a somewhat primitive and chaotic system which is structurally and functionally abnormal when compared to the host vasculature from which it develops [5]. As a result, areas form within the tumour which become oxygen deficient, nutrient deprived and highly acidic [5]. These adverse microenvironmental conditions not only play a significant role in influencing tumour response to conventional therapy [5,7], they also influence malignant progression in terms of making the primary tumour more aggressive and increasing its metastatic potential [7,8].

The importance of the tumour neo-vasculature in all these aspects makes it an excellent target for therapy and two major therapeutic approaches have now emerged [9,10]. One focuses on controlling tumour blood vessel development by inhibiting angiogenesis. Various angiogenesis inhibitors (AIs) have now been identified, each affecting at least one of the several important stages of angiogenesis. The principal targets are the angiogenic factors, which play the most significant role in neo-vascularization [11], especially vascular endothelial growth factor (VEGF), which is essential for endothelial cell proliferation and blood vessel formation [11,12]. The AIs that target VEGF include monoclonal antibodies and inhibitors of endothelial cell receptor-associated tyrosine kinase activity [13,14,15,16]. Other approaches target the various physical steps in the angiogenesis process including basement membrane degradation, endothelial cell migration, endothelial cell proliferation, and tube formation [13,14,15,16]. Many of these anti-angiogenic therapies have undergone clinical evaluation [13,15,17,18]. The other major approach for targeting tumour vasculature involves compromising the function of the already established tumour blood vessels using so called vascular disrupting agents (VDAs). These include physical treatments like hyperthermia, photodynamic therapy (PDT) and even radiation; biological response modifiers; cytokines; certain chemotherapeutic drugs; and various ligand-based approaches that selectively bind to tumour vessels [10,19,20,21,22]. However, the approaches that have received the greatest attention involve small molecule drugs [22]. These include flavonoid derivatives that target endothelial cells through a cascade of direct and indirect effects that include the induction of cytokines leading to the induction of haemorrhagic necrosis [23,24]. It also includes tubulin-binding agents that selectively disrupt the cytoskeleton of proliferating endothelial cells, resulting in endothelial cell shape changes and subsequent thrombus formation and vascular collapse [24]. Both types of small molecular drugs have been shown to have potent anti-vascular and anti-tumour efficacy in a wide variety of preclinical models and the lead agents have also undergone clinical evaluation [20].

### 1.2. *In vivo* Imaging of Antivascular Treatments

The inadequate vasculature is the main cause of the disadvantageous tumour microenvironment, and information about the vasculature has been shown to be of prognostic value. Microvascular density (MVD) as an estimate of angiogenesis has been shown to be prognostic of metastatic disease and overall and disease specific survival in some malignant diseases [25,26,27]. Angiogenic factors, especially VEGF, have also been shown to be prognostic. VEGF has been significantly correlated with disease-specific survival in patients with various cancers, for example prostate cancer [28] and breast cancer (for review concerning the latter, see [29]).

The above assays are immunohistochemical, and noninvasive *in vivo* assays are hence desirable for prognosis assessment and therapeutic guidance. Noninvasive vascular information is of further relevance in evaluating antivascular treatments as they have the vasculature as the main target. Many immunohistological methods (e.g., MVD based on endothelial staining) do not take into account that a varying degree of tumour neovasculature is non-functional [30]. Whether information on all vasculature, functional vasculature, or both is important depends on the particular study. Assessing functional vasculature has been done using functional vascular staining and radiolabelled iodoantipyrine (IAP). Microenvironmental changes induced by vascular disrupting agents may indeed potentiate the clinical outcome when combined with other treatments. Imaging techniques may detect vascular changes and provide evidence of this beneficial action. This noninvasive vascular information may also prove important in optimizing such treatments. In radiation therapy research, the suggested effect on vasculature [31,32] may also be evaluated by the same imaging modalities.

Several imaging modalities can provide *in vivo* information about vasculature [33]. These include dynamic methods in ultrasonography [34,35], computed tomography (CT) [36], positron emission tomography (PET) [37], bioluminescence imaging [35], and magnetic resonance imaging (MRI). MRI has been proven useful as a noninvasive and nonionizing method for acquiring information about tumour microvasculature, and it offers several different methods for obtaining vascular information. Issues regarding the use of MRI in assessing the clinical effect of vascular targeting treatments are discussed in [38]. The MRI methods include arterial spin labelling (ASL), dynamic susceptibility contrast MRI (DSC-MRI), blood oxygenation level dependent (BOLD) imaging, and dynamic contrast enhanced MRI (DCE-MRI). ASL does not involve use of contrast agent, but it relies on a small signal and provides only perfusion information [39,40,41]. DSC-MRI uses first-pass bolus tracking of contrast agent [42,43]. This method gives information about relative perfusion, blood volume, and mean transit time. It is possible to also estimate average vessel size [44]. The susceptibility contrast relies on the contrast agent remaining intravascular, and the method is mostly used in the brain where the blood-brain-barrier prevents extravasation of small contrast agents. Both ASL and DSC-MRI give relative measures. A static susceptibility contrast approach is estimation of the transverse relaxation times T_2_ and T_2_^*^ before and after injecting a high-molecular-weight contrast agent, which acts as a blood pool agent (BPA) remaining intravascular at a constant concentration. This strategy allows estimation of relative blood volume and average vessel size [45,46]. Susceptibility contrast information on functional blood volume can also be obtained by BOLD by manipulating levels of endogenous deoxygenated haemoglobin, which is paramagnetic like the mentioned contrast agents [30].

DCE-MRI is widely used in both preclinical and clinical oncology for obtaining vascular information, and it is easily implementable on most MR scanners. DCE-MRI parameters have been correlated both with immunohistochemical assays and with outcome, for a review of studies, see [47]. Because of its extensive use in preclinical assessment of antivascular treatments, DCE-MRI was chosen as imaging modality for this review.

## 2. DCE-MRI

### 2.1. Principle

DCE-MRI is based on dynamic T_1_-weighted imaging of contrast agent extravasation over several minutes often with lower time resolution than needed to track a first-pass bolus. Often, a fast spoiled gradient echo sequence is used. Historically, increased blood vessel permeability in brain lesions and tumours can be detected by static imaging of contrast agent accumulation in a time window following contrast agent administration [48,49,50]. Analysis of dynamic data provides additional information. Many analysis approaches and parameters exist, but extravasation, interstitial volume, and sometimes blood volume are the main information obtained. The extravasation information depends on both perfusion and permeability, and separation of these parameters is difficult. However, this is a novel research area [51,52,53].

### 2.2. Semiquantitative Analysis

The dynamic data can be analysed either voxel-wise, or the average signal in a region of interest (ROI) can be calculated for analysis. Analysis methods can be separated into semi-quantitative approaches or quantitative models. From signal-time curves, semi-quantitative parameters like initial area under the curve (IAUC) for 60 or 90 s, maximum signal enhancement, and time-to-peak can be calculated. With correctly chosen sequence parameters, the relative signal is linearly proportional to the contrast agent concentration, which facilitates comparison between studies [54]. The signal-time curves can also be converted to concentration-time curves if the contrast agent’s relaxivity *r*_1_, *i.e.*, the ability to shorten relaxation time T_1_, is known and the initial T_1_ is estimated or another calibration is performed. This assumes a linear relaxivity and fast water-exchange between tissue compartments. Corresponding semi-quantitative analysis can be done on concentration-time-curves, but the parameters then involve quantitative concentrations. IAUC from concentration-time curves is a simple and robust estimate of vascularisation, and it is easily comparable between studies. However, the initial signal originates from several physiological properties including blood volume, perfusion, vessel permeability, and interstitial volume.

### 2.3. Model Analysis

The model analyses attempt to separate different physiological information from the concentration-time curves. Different models have been utilized, and often they involve freely diffusible tracer kinetics developed for non-imaging methods [55,56,57,58]. Tofts *et al.* compared three different models [59] by Tofts and Kermode [60], Larsson *et al.* [61], and Brix *et al.* [62] and discussed their assumptions and differences in the information they provide. Also the Patlak plot has been utilized for DCE-MRI analysis [63]. A consensus kinetic two-compartment model based on the model by Tofts and Kermode with standardization of parameters and units has been published by leading researchers [64]. The model describes the transport of contrast agent between the blood plasma and extravascular extracellular compartments, and it estimates the volume transfer constant between blood plasma and extravascular extracellular space, *K*^trans^, the rate constant between extravascular extracellular space and blood plasma, *k*_ep_, and the volume of extravascular extracellular space per unit volume of tissue, *v*_e_ = *K*^trans^/*k*_ep_ by fitting the obtained contrast-time-curves to the model Equation:

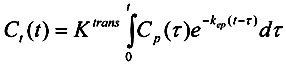
(1)
where *C*_t_(t) is the tissue concentration of Gd-DTPA, and *C*_p_(t) is the plasma concentration of Gd-DTPA. The model is often extended to also estimate *v*_p_, which is otherwise assumed to be zero:

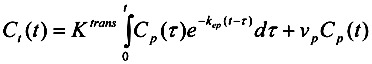
(2)

The Patlak model has been extended to also include the transfer rate from the extravascular extracellular compartment to the plasma compartment, and this model is similar to the extended Toft’s model (Equation 2) [65,66]. *C*_p_(t) can be measured from the dynamic images, but this is not trivial because of partial volume-effects and inflow-effects [67]. Tofts and Kermode [60] have utilized an assumed human input function, which is a model fit to experimental clinical data by Weinmann *et al.* [68] Similar model input functions exist for animal models, for example rats [69,70] and mice [71]. Another approach is the use of reference tissue in the dynamic images [72,73].

### 2.4. Contrast Agents

Differently sized contrast agents are used for DCE-MRI. The low-molecular Gd-DTPA leaks from physiological blood vessels outside the brain and to a larger extent from hyper-permeable tumour blood vessels. BPAs remain intravascular in healthy tissue, but often extravasate in tumours, however at a longer time-scale than low-molecular contrast agents. Blood vessel permeability is size-dependent such that both the vessel pore size and the contrast agent size influence the permeability and perfusion contributions to extravasation. Therefore, DCE-MRI parameters may reflect different physiology with different sensitivity dependent on the contrast agent size [74,75,76]. Often, BPAs remain intravascular in tumours for several minutes allowing susceptibility MRI as well as DCE-MRI over a longer time scale [77].

## 3. Antiangiogenic Treatments

### 3.1. Antibodies and Specialized Proteins

We have separated the AIs into three subgroups based on their mechanism of action. They are presented in Table 1.

**Table 1:** Angiogenesis inhibitors.

Compound	Angiogenic target	Schedule/dose	References
*Antibodies and specialized proteins*			
Bevacizumab *	VEGF-A (VEGF_165_)	1–6 × 5–45 mg/kg	[78,79,80,81,82,83,84,85,86,87,88]
DC101	VEGFR-2	7 × 30mg/kg	[89]
VEGF-trap	VEGF-A/B, PlGF	4 × 25mg/kg	[90]
*Tyrosine Kinase Inhibitors*			
Vatalanib	PDGFR-β, c-kit, VEGFRs	7–14 × 50–100 mg/kg	[91,92,93,94]
Sunitinib	VEGFRs, PDGFRs	1–7 × 40–45 mg/kg	[95,96]
Orantinib	VEGFR-2, c-kit, FGFR, PDGFR	1–14 × 200 mg/kg	[97,98]
Vandetanib	VEGFR-2, EGFR	2 × 12.5–100 mg/kg	[51,99]
Axitinib	PDGFR, c-kit, VEGFR-1/2/3	14 × 25 mg/kg	[100]
Imatinib	PDGFR-β, c-kit, abl, VEGFR-2	3 × 50 mg/kg	[101]
Cediranib	VEGFRs	1–20 × 6 mg/kg	[102]
Sorafenib	VEGFRs, PDGFRs, Raf	25 × 7 mg/kg	[103]
*Others*			
TNP-470	MetAP2	3–7 × 6.7–30 mg/kg	[104]
Everolimus	mTOR	1–7 × 5–10 mg/kg	[105]
KR-31831	Unknown	21 × 50 mg/kg	[106]
Thalidomide	FGF-2	2–3 × 60–200 mg/kg	[107,108,109]

* and other anti-VEGF antibodies.

The first subgroup comprises monoclonal antibodies and specialized proteins that interfere with the extra-cellular signalling molecules by targeting soluble growth factors or mimicking growth factor receptors.

Among these bevacizumab (and other anti-VEGF antibodies) is by far the most used in the clinic and the best described angiogenesis inhibitor in the literature [17]. In preclinical setups where DCE-MRI is performed to evaluate the anti-vascular effects of bevacizumab, all experiments were performed in immuno-supressed rats (athymic nude). These were bearing either VEGF-expressing human breast cancers [78,79,82,83,85], human glioblastomas [81,84,87], a human ovarian cancer [80], or a small cell lung carcinoma [86]. Treatment dose and schedule varied among experiments as did the imaging schedule (before and from 1 to 30 days after treatment initiation), contrast agent (gadopentetate dimeglumine (Gd-DTPA), Albumin-Gd-DTPA, gadodiamide (Gd-DTPA-BMA), gadoteridol (Gd-HP-DO3A), and gadocoletic acid) and dose of contrast agent (0.03 to 0.50 mmol Gd/kg). Despite the many variations in experimental setup all investigations reported a consistent decrease in tumour vascular permeability, as estimated from K(PS) or AUC. A study by Fruth *et al.* [88] used a mixture of anti-VEGF antibodies to compensate for radiation-induced VEGF-production. Treatment and imaging circumstances were similar to the bevacizumab experiments except the use of mice with a squamous cell carcinoma. DCE-MRI indicated reduced tumour blood volume and hence constrained actions of VEGF. However, they also observed an increase in basic fibroblast growth factor- (bFGF-) production as yet another survival mechanism of the tumour cells.

Another approach at inhibiting the VEGF-VEGFR interaction was taken by Kiessling *et al.* [89] and Hoff *et al.* [90] who used DC101 and VEGF-trap, respectively. Although very different setups were used the consensus was still a decrease in tumour vascularization defined by DCE-MRI parameters.

The majority of experiments in this subgroup were validated using histology to assess vascular morphology, vessel density or cell viability/necrosis.

### 3.2. Tyrosine Kinase Inhibitors

The second major subgroup of angiogenesis inhibitors are the Tyrosine Kinase Inhibitors (TKIs). These can specifically inhibit the phosphorylation of tyrosines and hence interrupt the downstream signalling cascade of activated growth factor receptors. This inhibition is achieved by either competing with adenosine triphosphate (ATP), competing with the substrate protein that is to be phosphorylated, or by conformational change leading to allosteric inhibition. The majority of TKIs are based on competitive binding in or around the active ATP-binding site of the tyrosine kinases. Most TKIs target the VEGF signalling pathway, and many TKIs target multiple growth factor receptors, leading to multiple shut down of signalling pathways.

Vatalanib (PTK787/ZK222584) is the most investigated drug in preclinical setups using DCE-MRI to evaluate anti-angiogenic effects. Both mice and rats have been used for *in vivo* models to test vatalanibs effect on a range of tumour xenografts/cell lines (human breast cancer [91], melanoma cells [92], glioma cells [93], and renal cell carcinoma [94]). The treatment dose and schedule for these experiments are somewhat similar (table), and the same can be said about the use of contrast agents (gadoterate meglumine (Gd-DOTA) or albumin-Gd-DTPA) and imaging schedules (imaged before and after last treatment). In three of four tumour models a decrease in vascular permeability was observed as estimated from *K*^trans^ and K(PS). The only exception was the glioma model that showed an increase in *K*^trans^ in the tumour periphery. This difference could be explained by the lack of tumour segmentation when analysing the DCE-MRI data. In the three studies that showed a decrease in permeability the tumour ROI was not segmented, whereas the fourth study had divided the tumour into a core and a rim region. The lack of segmentation could hence camouflage a local peripheral increase in permeability, and explain the observed differences. Ali *et al.* [93] found that the increase in permeability of the tumour rim was attributed to vasodilation due to VEGF expression. 

Sunitinib (SU11248) was tested in mice bearing either a human colon carcinoma [95] or a pancreatic carcinoma [96]. Both experiments showed a decrease in *K*^trans^ although contrast agents (albumin-Gd-DTPA and P846), treatment schedules and hence imaging schedules were different.

In two very similar experiments [97,98] treatment with orantinib (SU6668) in mice bearing a human colon carcinoma revealed an enhancement in the tumour periphery after 3 days of treatment. This was however preceded by a decrease in permeability of both tumour rim and periphery 24 h after treatment. These observations indicated that prolonged treatment facilitates vascularization/vasodilation/permeabilization in the tumour periphery possibly due to increased expression of VEGF. This phenomenon was also observed in a study using vatalanib [93].

Vandetanib (ZD6474) and its less soluble predecessor ZD4190 were evaluated in mice bearing a human prostate cancer. Both studies used same treatment schedule and dose as well as imaging schedules. The only differences were the use of contrast agent, which was either a small agent [99] or a macromolecular agent [51], or the model used to analyse DCE-MRI data (de Bazelaire *vs.* Tofts). Vandetanib showed a dose-dependent decrease in *K*^trans^, which could be due to both perfusion and permeability as this study used the small contrast agent, and ZD4190 showed a reduction of tumour vascular permeability, vascular volume and blood flow. Both studies showed similar effects of anti-angiogenic treatment, but one should keep in mind that the size of contrast agent has major influence on the interpretation of the results. Macromolecular contrast agents are more likely to report permeability than perfusion, and *vice versa* for small contrast agents, but it will always be a combined measurement of the two parameters.

Axitinib (AG013736) [100], imatinib (STI571) [101], and cediranib (AZD2171) [102] were only used in one study each, involving mice bearing either a human breast cancer (axitinib), a human prostate cancer (imatinib), or different glioblastomas (cediranib). All studies used similar imaging schedules and contrast agents and showed a decrease in vascular permeability estimated either by *K*^trans^ or K(PS).

Last study in this subgroup involved sorafenib [103] treatment in rats bearing a human breast cancer. Reduced tumour blood flow and vascular permeability was achieved in this study as well.

To summarize, most TKIs induce a decrease in vascular permeability and perfusion, but interpretation should be done with caution as tumour segmentation, use of contrast agent, and prolonged treatment has an influence on the acquired DCE-MRI data. Indeed, this was addressed in the majority of studies in this subgroup as obtained results with DCE-MRI were supported by relevant histology.

### 3.3. Other Mechanisms of Action

This subgroup comprises drugs that exert their anti-angiogenic effect by targeting the endothelium (TNP-470 (AGM-1470)) [104], suppressing the immune system (everolimus (RAD001)) [105] or whose mechanism of action has not yet been clearly defined (KR-31831) [106].

TNP-470 is a fumagilin-analogue (antibiotic) that targets and irreversibly inactivates methionine aminopeptidase-2 (MetAP2) in endothelial cells. This results in inhibition of endothelial cell proliferation and migration, and hence tumour angiogenesis. This study used mice bearing a rat prostate cancer that were treated for approximately a week and imaged using a macromolecular contrast agent before and after the last treatment. This study observed a decrease in tumour vascular volume, as well as local increases and decreases in permeability.

Everolimus is an immuno-suppressant that, when in complex with the immunophilin FK binding protein-12 (FKBP-12), binds to and inhibits the mammalian target of rapamycin (mTOR). Evidence suggests that mTOR is an important signalling molecule in the activation of tumour angiogenesis [110]. In this study mice bearing B16/BL6 melanoma lymph node metastasis and rats bearing BN472 mammary tumours were imaged with both small and macromolecular contrast agents before and after treatment. Despite an observed anti-tumour effect of everolimus, no significant difference was observed in any of the DCE-MRI parameters, except a reduced Ve in mice bearing B16/BL6. Authors attributed this to the devastating effect of everolimus on both pericytes and endothelial cells. By destroying the covering pericytes, vascular leakage would still be in effect and give no change or even increase the *K*^trans^ parameter. 

The drug KR-31831 has been reported to suppress endothelial cell proliferation, tube formation, invasion, and migration *in vitro* as well as vessel formation *in vivo* [106]. The mechanism of its anti-angiogenic actions remains unclear but is indicated to interfere with the VEGF signalling pathway. In this study mice bearing a human ovarian carcinoma were imaged using a small contrast agent and a decrease in *K*^trans^ was observed from day 0 to day 21 in treated animals. No difference in *K*^trans^ was observed in controls.

Thalidomide has been investigated in different scenarios [107,108,109]. One study found reduced vessel permeability for a macromolecular contrast agent as a result of vascular normalization [108], while another study found increased blood plasma volume fraction [107].

As this subgroup contains drugs that have different mechanisms of action it is not surprising to find that they behave differently when analysed with DCE-MRI. Another lesson to take from these studies is that if a drug has multiple or unknown effector functions these can counteract or influence each other giving unexpected results when DCE-MRI parameters are estimated.

## 4. Vascular Disrupting Agents

### 4.1. Tubulin Binding Agents

We have separated the VDAs into three subgroups based on their mechanism of action. They are presented in Table 2.

**Table 2:** Vascular disrupting agents.

Compounds	Dose/schedule	DCE-MRI	References
**Tubulin binding**			
CA4P	1-2 × 10-250 mg/kg (mouse)	1-24 h	[77,111,112,113,114,115,116,117,118,119,120,121]
	1 × 10-100 mg/kg (rat)	1 h-9 days	[70,122,123,124,125]
OXi4503	1-2 × 25-100 mg/kg (mouse)	4-144 h	[114]
ZD6126	1 × 50-200 mg/kg (mouse)	24 h	[126,127]
	1 × 2.5-50 mg/kg (rat)	1-120 h	[125,128,129,130,131]
NPI2358	1 × 2.5-15 mg/kg (mouse)	1-24 h	[132]
Stilbene 5c and 6c	1 × 50 mg/kg (mouse)	4 h	[133]
TZT-1027	1 × 0.5 mg/kg (rat)	1-3 h	[134]
ABT-751	1 × 30 mg/kg (rat)	1-6 h	[135]
**TNF-α inducing**			
DMXAA	1-4 × 22-30 mg/kg (mouse)	3-24 h	[113,136,137,138,139,141,142,143]
	1 × 100-350 mg/kg (rat)	4-24 h	[144]
TNF-α	300 μg/kg or viral overexpression (mouse)	2-96 h or 3 days	[145,146]
AP/1649, AP/1897	4 × 27 mg/kg (mouse)	3-24 h	[143]
**Other**			
Radiation	8-18 Gy single dose or fractionated (rat)	2-25 days	[147,148]
	20 Gy single dose or fractionated (mouse)	3-120 h	[32]
*Photosensitizers*			
Bacteriochlorophyll-serine	1 × 20 mg/kg (mouse)	1-24 h	[136,149]
*Targeted chemotherapy*			
MBT-0206	3 × 5 mg/kg (hamster)	24 h	[150]
EndoTAG^®^-2	6 × 2.5 mg/kg (mouse)	24 h	[151]

**Figure 1:**
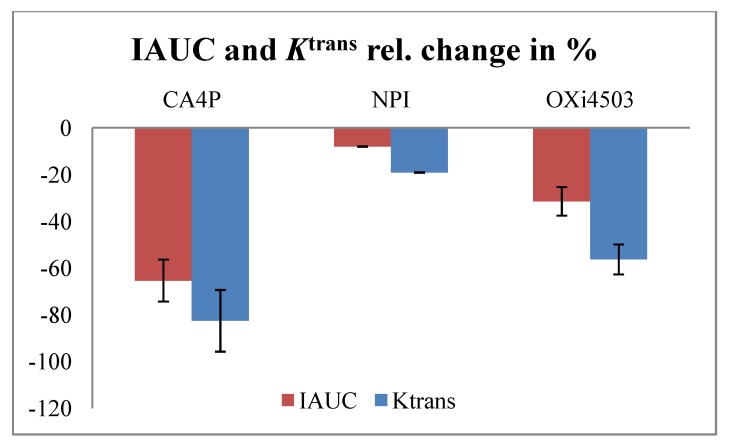
Effect of combretastatin A-4 phosphate (CA4P) (25 mg/kg) [117], NPI (7.5 mg/kg) [132], and combretastatin A-1 Phosphate (OXi4503) (50 mg/kg) (unpublished data) in 200 mm^3^ C3H mammary carcinomas as measured by initial area under the curve (IAUC) (90 s) (red) and *K*^trans^ from Toft’s model (blue) 3 h following treatment. Bars show mean of tumour median values ± SE.

For a recent review of clinical studies and DCE-MRI considerations, see [152]. Tubulin binding agents depolymerises tubulin or inhibits polymerization of tubulin to microtubules, which are part of the cytoskeleton maintaining the shape of endothelial cells. The induced changes in endothelial morphology cause vascular shut-down within a few hours and induce necrosis detectable at 24 h. The tubulin binding agents include combretastatins, which are natural stilbenoid natural phenols isolated from South African Bush Willow *Combretum caffrum*. Studies include the derivatives combretastatin A-4 phosphate (CA4P) and combretastatin A-1 Phosphate (OXi4503 (CA1P)). ZD6126 is a prodrug of *N*-acetylcolchinol, related to colchicines. NPI2358 (plinabulin) has been derived from a marine microbial source. It binds to the colchicine binding site of b-tubulin preventing polymerization and disrupting the cytoplasmic microtubule network. Other agents are stilbene 5c and 6c, TZT-1027 (soblidotin), and ABT-751. The different effect of CA4P, OXi4503, and NPI2358 in the C3H mammary carcinoma is shown in Figure 1. IAUC and *K*^trans^ from concentration time curves and Toft’s model showed the same effect.

**Figure 2:**
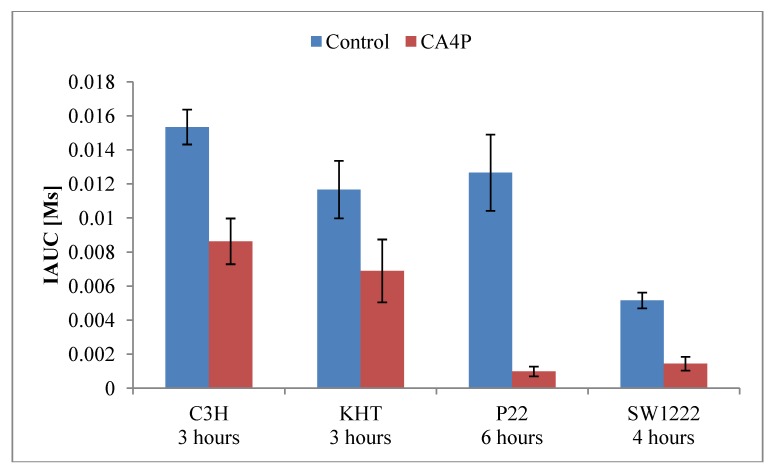
Effect of CA4P (100 mg/kg) as measured by IAUC (90 or 94 s from concentration time curves) following injection of gadopentetate dimeglumine (0.1 mmol/kg) in 4 tumour models: C3H mammary carcinoma [117], KHT sarcoma [117], P22 carcinosarcoma [70], and SW1222 human colonic adenocarcinoma [115]. Bars show mean of tumour mean or median values ± SE.

DCE-MRI studies of CA4P has been done in the rat tumour models P22 carcinosarcoma[70,122], 13762NF mammary carcinoma[123], and R1 liver rhabdomyosarcoma[124,125], and in the murine tumour models sarcoma F [111], RIF-1 fibrosarcoma [112], SaS sarcoma [112], SaF sarcoma [112], C3H mammary carcinoma [77,112,117,119,120], KHT sarcoma [114,116,119,120], and EL4 lymphoma [121]. In immunodeficient mice, the following human tumour models were investigated; the colon adenocarcinomas HT29 [112,113], LoVo [112], LS174T [113], and SW1222 [115], and the mammary carcinoma MDA-MB-231 [118]. The CA4P doses investigated were single injections in the dose range 10–100 mg/kg in rats, and single or two injections of 10–250 mg/kg in mice. Imaging schedules varied with DCE-MRI examinations before and from 1–144 h following treatment, and the contrast agents included gadopentetate dimeglumine (0.1-0.3 mmol/kg), gadodiamide (0.04–0.2 mmol/kg), gadoterate meglumine (0.2 mmol/kg), and feruglose (NC100150) (2.5 mg Fe/kg). The effect of a single CA4P dose (100 mg/kg) on IAUC in 4 different tumour models is shown in Figure 2.

**Figure 3:**
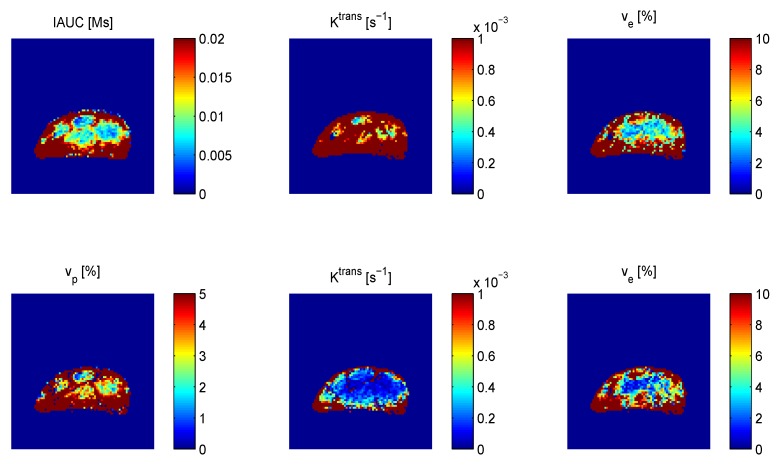
Parametric maps of IAUC (90 s) and parameters from Toft’s model excluding (**upper**) and including (**lower**) blood volume fraction *v*_p_ in a 200 mm^3^ C3H mammary carcinoma.

In general, the studies show reduction in perfusion and permeability 1–6 h after treatment. Changes in IAUC and *K*^trans^ tended to be smaller than measured by radiolabelled IAP, but have shown similar dose-response and time course [70,122]. Some studies found dependency on tumour model [112,113,119]. Beauregard *et al.* [112,113] found that decrease in energy status by ^31^P nuclear magnetic resonance (NMR) spectroscopy corresponded well with DCE-MRI changes in a range of tumour models. Zhao *et al.* found consistent results from DCE-MRI and bioluminescence imaging [118]. Often, histology has been used to assess necrosis, which is typically seen in the tumour centre from 24 h after treatment. Also histology involving vascular staining and the functional perfusion marker Hoechst 33342 was often used to support the DCE-MRI data. Perfusion recovery was seen in the periphery or whole tumour from 24 h to 9 days [114,115,121,123,124,125]. Recovery in the tumour rim while central perfusion remained low has been correlated with oxygen levels by ^19^F oximetry [123]. One study in the KHT sarcoma compared CA4P and the other combretastatin derivative OXi4503 showing a slower perfusion recovery after OXi4503 treatment [114]. A dose response of CA4P in the C3H mammary carcinoma was similar in shape to a dose response of CA4P in combination with radiotherapy as estimated by TCD_50_ indicating that the vascular effects 3 h after treatment reflect the drug’s impact on radiotherapy [117]. An optimal dose was found at 25 mg/kg, and the doses were converted to milligram per square millimeter surface for comparison with a clinical study. At this optimal dose, *K*^trans^ from the Toft’s model was reduced by 83%, but if the model was extended to estimate blood plasma volume *v*_p_, *K*^trans^ was reduced by 47% and *v*_p_ by 81% indicating that blood volume change was a part of the response. Figure 3 shows that *K*^trans^ was lower when *v*_p_ was estimated rather than assumed negligible.

ZD6126 has been investigated in the rat tumour models GH3 prolactinoma [128,129] and R1 rhabdomyosarcoma [125,131], and the Hras5 transformed NIH 3T3 fibroblast in athymic rats [130]. The C38 colon adenocarcinoma was investigated in mice [126], and a study on the DU-145 human prostate cancer was done in severe combined immunodeficient (SCID) mice [127]. In rats, doses between 2.5–50 mg/kg were given, and in mice doses varied from 50–200 mg/kg. DCE-MRI was performed before and 1–120 h following single drug injections, and the contrast agents used were gadopentetate dimeglumine (0.3 mmol/kg), gadodiamide (0.1 mmol/kg), gadoterate meglumine (0.02 mmol/kg), and albumin-Gd-DTPA (500 mg/kg). The studies show vascular shutdown in a dose-dependent manner with slightly different time courses seen in the rat studies, which may depend on drug dose and tumour type. Using low-molecular weight contrast agents (gadodiamide and gadoterate meglumine), Wang *et al.* found *K*^trans^ to be decreased at 1 h with recovery starting from 6 h (dose 10 mg/kg) [125] or 48 h (dose 20 mg/kg) [131] in the R1 rhabdomyosarcoma, and McIntyre *et al.* found no signs of recovery up to 96 h after treatment with the dose 50 mg/kg in the GH3 prolactinoma [129].

NPI2358 given at doses ranging from 2.5 to 15 mg/kg was tested by DCE-MRI with gadopentetate dimeglumine in the mouse C3H mammary carcinoma [132]. At a dose of 7.5 mg/kg, NPI2358 reduced IAUC and *K*^trans^ after 1 h, peaked at 3 h and returned to normal after 24 h. When extending Toft’s model to estimate *v*_p_, *v*_p_ was more likely to change significantly than *K*^trans^ indicating that blood volume change was a part of the response.

Stilbene 5c and 6c are also microtubule inhibitors binding at the colchicine binding site. They have been tested in the human ovarian cell cancer line UCI-101/luciferase at a single dose of 50 mg/kg [133]. DCE-MRI with 50 μL gadodiamide showed selective suppression of tumour perfusion without damaging normal organ perfusion.

TZT-1027 is another tubulin inhibitor with cytotoxic properties used in chemotherapy. Its antivascular properties at a single dose of 0.5 mg/kg has been tested by DCE-MRI with gadopentetate dimeglumine (0.2 mmol/kg) in the human breast tumour MX-1 (grown in mouse) and DiMethylBenz(a)Anthracene induced breast tumours (grown in rats) showing reduced blood flow 1–3 h after treatment in both tumour types [134].

The novel tubulin-binding agent ABT-751 has been investigated by DCE-MRI using gadopentetate dimeglumine (0.2 mmol/kg) in the 9 L glioma in rats [135]. A single injection at the dose 30 mg/kg resulted in reduced tumour perfusion after 1 h and restoration of perfusion after 6 h. Perfusion was estimated as the maximum slope of signal intensity curves, and the effect was seen in all of 3 tumour regions defined by their distance to the tumour edge.

### 4.2. TNF-α Inducing Agents

The tumour necrosis factor-α (TNF-α) inducing drug DMXAA (Vadimezan, ASA404) has been investigated by DCE-MRI in the rat GH3 prolactinoma [144], in a range of murine tumours including MCA205 (methylcholantrene-induced fibrosarcoma) [139] and SaF sarcoma [143], and in a number of human xenografts in mice including HT29 and LS174T colon adenocarcinomas [113], FaDu [137,141,142], A253 [137], and patient-derived [142] head and neck carcinomas. Seshadri *et al.* used different Gd-based macromolecular contrast agents to assess blood volume and permeability [137,139,141,142]. Additional MRI studies by this group assess extravasation and accumulation of macromolecular contrast agent at a longer time scale, 4 and 24 h, in the murine Colon-26 colon carcinoma [136] and CT-26 colon adenocarcinoma [138], and in the human U87 glioma [140]. In rats, doses in the range 100–350 mg/kg were used, and in mice doses varied between 22–30 mg/kg. DCE-MRI was scheduled before treatment or in control animals, and from 3–24 h after treatment using the contrast agents gadopentetate dimeglumine (0.2 mmol/kg), gadodiamide (0.1 mmol/kg), gadofosveset (Gd-DTPA derivative) (0.1 mmol/kg), albumin-Gd-DTPA (0.1 mmol/kg), poly-lysine-Gd-DTPA (0.1mmol/kg), or methoxy-PEG succinyl-poly-L-lysine-GdDTPA (0.1mmol/kg). The main findings are vascular disruption 24 h following treatment seen with decreased IAUC or *K*^trans^. Increased permeability 4 h after treatment followed by decreased permeability 24 h after treatment has been observed in two studies [136,138]. Also marked extravasation and accumulation of albumin-Gd-DTPA 24 h following treatment indicated vascular disruption [140]. In the rat GH3 prolactinoma [144], an effect was only seen with the large dose of 350 mg/kg.

In the study of Barbera *et al.* [143], the DMXAA analogues AP/1649 and AP/1897 showed no antivascular effect in the SaF sarcoma as seen by DMXAA. Single injection of TNF-α at a dose of 300 μg/kg in the MC38 murine colon adenocarcinoma showed reduced vascular parameters from 2–96 h following treatment. Tumours infected with Ad.EGR-TNF respond to radiation with induction of TNF-α expression, and this was investigated in the PC-3 human prostate cancer grown in athymic mice [146]. DCE-MRI and electron paramagnetic resonance imaging (EPRI) suggested reduced perfusion and oxygenation 3 days after treatment.

### 4.3. Other Vascular Disrupting Treatments

The antivascular effects of radiotherapy have been investigated by DCE-MRI in a few studies [32,147,148]. In the MAT-LyLu tumour (subline of the Dunning R3327 rat prostate adenocarcinoma), Kiessling *et al.* [147] found the irradiation slowed tumour growth, but no change in the DCE-MRI parameter Kep from the Hoffman model using gadopentetate dimeglumine (0.2mmol/kg). The lack of difference could be explained by the rapid regeneration of the MAT-LyLu tumour subtype. De Kayser *et al.* 1090 [148] compared single dose 8 Gy with 5 × 3 Gy in the R1 rhabdomyosarcoma. Single dose radiation showed perfusion decrease 2 days after treatment, with a slow recuperation. Fractionated radiation showed similar perfusion decrease early after treatment. Horsman *et al.* [32] also compared single dose *vs.* fractionated irradiation in the C3H mouse mammary carcinoma. A large single dose was superior to the same dose given in a more conventional fractionated schedule, but vascular-mediated effects did not account for this.

Photosensitizers also have vascular disrupting properties. Zilberstein *et al.* [149] found that a single injection of bacteriochlorophyll-serine at the dose 20 mg/kg in a study of the M2R melanoma xenograft in mice caused severe reduction in tumour perfusion 24 h after treatment. Seshadri *et al.* [136] found that photodynamic treatment with 2-[1-hexyloxyethyl]-2-devinyl pyropheophorbide-a (HPPH) (0.4 µmol/kg) or 5-aminolevulinic acid (ALA) (6 × 20 µL) produced subtle vascular injury below the threshold needed to achieve the catastrophic vascular collapse and dissolution. Combination of PDT and DMXAA provided therapeutically synergistic and selective antitumor activity.

Chemotherapy targeted against the vasculature has been investigated by Eichhorn *et al.* [150,151]. Paclitaxel, a tubulin stabilizer, encapsulated in cationic lipid complexes (MBT-0206) was administered at an effective dose of 5 mg/kg daily for 3 days in the amelanotic hamster melanoma A-MEL-3 [150]. 24 h after last treatment, a decrease in intratumoural blood volume and increase in vascular permeability were seen. The topoisomerase I inhibitor camptothecin in cationic lipid complexes (EndoTAG-2) administered at the effective chemotherapy dose 2.5 mg/kg on days 7, 9, 11, 14, 16, and 18 after inoculation of the Lewis Lung Carcinoma LLC-1 in mice also gave significant reduction in tumour vascular volume and tumour perfusion [151].

## 5. Conclusions and Future Perspectives

DCE-MRI is a useful tool to assess *in vivo* drug effects on vasculature. The term DCE-MRI covers methods with different imaging sequences, analysis of either (relative) image signal time curves or concentration time curves with different descriptive or model based parameters. The parameters are furthermore influenced by the choice of contrast agent. While this amount of possibilities makes it possible to tailor DCE-MRI methods to individual studies, it makes study comparison challenging. One needs to have the actual method in mind when reading a published study. Attempts have been done to standardize methods and nomenclature [54,64], and especially in clinical trials standardization is important. Preclinical studies should be comparable as well, but they also benefit from a larger arsenal of contrast agents, magnet field strengths, and additional imaging and histological methods. In this review, we have to a large degree maintained the individual papers’ terminology and interpretation and at some places commented on relevant method details.

A general and returning issue is the dependence of *K*^trans^ and other extravasation-describing parameters on both perfusion and permeability. Reduced perfusion occurring simultaneously with increased permeability surface area product as a result of vascular targeting will influence extravasation in opposite ways. The extravasation-describing parameter would in this case provide little and hardly interpretable information on the microvascular situation. As stated in the introduction, separation of these properties is not trivial and part of the standard models. Another influence on *K*^trans^ is blood volume when an invalid assumption of negligible blood volume fraction is made. The initial fast increase in concentration time curves arising from the filling of blood vessels will in this case be attributed to extravasation instead of blood volume. Figure 3 shows an example of difference in *K*^trans^ when comparing Toft’s model analysis without and with inclusion of a vascular term (Equations 1 and 2). This illustrates the effect seen in [117] where the CA4P effect on *K*^trans^ was lower when *v*_p_ was estimated.

DCE-MRI analysis is sometimes made in ROIs covering whole or part of the tumour, but very often the analysis is made voxel-wise. In studies with voxel-wise analysis, a single value such as median or mean is often used for statistics. The median can be chosen to avoid heavy influence on the mean value from single outlier estimates from e.g. poor curve fits. Preclinical studies are often performed at higher magnetic field strengths allowing higher spatial resolution. This could provide insight into partial-volume effects at clinical field strengths. In voxel-wise analyses, the spatial information can be described statistically, and a tumour’s voxels can be segmented into groups. Tumours lack anatomical fix points, and segmentation can be done by different principles. Some involve spatial knowledge, e.g., that VTAs often show different effects in the tumour centre *vs.* the tumour rim. A ROI can be drawn manually, by morphological image operations segmenting e.g., the tumour rim, or by region growing to expand a chosen area to include voxels with similar properties. Other segmentation methods assume no spatial knowledge, e.g., histogram thresholding or multivariate clustering. We have attempted to investigate the effect of CA4P in C3H mammary carcinomas segmented into three regions with similar characteristics based on the three parameters estimated from the extended Toft’s model [119]. The method did not take into account the voxels’ spatial location, but the identified regions tended to be connected rather than scattered. Segmentation may provide more information from DCE-MRI data, but the many approaches make study comparison even more difficult.

DCE-MRI is an important method for assessing the effect of antivascular treatments in preclinical studies. The imaging protocol and analysis involve many choices that can optimize the individual studies but make study comparison harder. A consensus model has been proposed to overcome this, but other models are still relevant. Future perspectives involve more complicated models separating perfusion and permeability, and also improved use of spatial image information.
